# The effects of collagen peptide supplementation on body composition, collagen synthesis, and recovery from joint injury and exercise: a systematic review

**DOI:** 10.1007/s00726-021-03072-x

**Published:** 2021-09-07

**Authors:** Mishti Khatri, Robert J. Naughton, Tom Clifford, Liam D. Harper, Liam Corr

**Affiliations:** 1 School of Human and Health Sciences, University of Huddersfield, Huddersfield, UK; 2 School of Sport, Exercise and Health Sciences, Loughborough University, Loughborough, UK

**Keywords:** Gelatin, Joint health, Joint pain, Protein, Fat free mass, Muscle damage

## Abstract

Collagen peptide supplementation (COL), in conjunction with exercise, may be beneficial for the management of degenerative bone and joint disorders. This is likely due to stimulatory effects of COL and exercise on the extracellular matrix of connective tissues, improving structure and load-bearing capabilities. This systematic review aims to evaluate the current literature available on the combined impact of COL and exercise. Following Preferred Reporting Items for Systematic Reviews and Meta-analyses guidelines, a literature search of three electronic databases—PubMed, Web of Science and CINAHL—was conducted in June 2020. Fifteen randomised controlled trials were selected after screening 856 articles. The study populations included 12 studies in recreational athletes, 2 studies in elderly participants and 1 in untrained pre-menopausal women. Study outcomes were categorised into four topics: (i) joint pain and recovery from joint injuries, (ii) body composition, (iii) muscle soreness and recovery from exercise, and (iv) muscle protein synthesis (MPS) and collagen synthesis. The results indicated that COL is most beneficial in improving joint functionality and reducing joint pain. Certain improvements in body composition, strength and muscle recovery were present. Collagen synthesis rates were elevated with 15 g/day COL but did not have a significant impact on MPS when compared to isonitrogenous higher quality protein sources. Exact mechanisms for these adaptations are unclear, with future research using larger sample sizes, elite athletes, female participants and more precise outcome measures such as muscle biopsies and magnetic imagery.

## Introduction

Collagen constitutes one-third of the total protein in humans and is the most abundant form of structural protein in the body. The primary role of collagen is to maintain connective tissue health and mechanical properties of the skin (Ricard-Blum 2011). As collagen is the principal component of the extracellular matrix (ECM), it is vital for the strength, regulation, and regeneration of this tissue (Frantz et al. 2010). Collagen also contributes ~ 65–80% dry weight of tendons, with collagen crosslinks aiding the tendon structure to endure resistance from high-impact stresses and shear forces (Kannus 2000). Thus, collagen plays a vital role in maintaining tendon health and mitigating potential injury-risk in sport (Goes et al. 2020).

Collagen is characterised by a high concentration of three amino acids—glycine, proline, and hydroxyproline, which create its characteristic triple-helix structure (León-López et al. 2019). Hence, collagen is hydrolysed enzymatically, degrading it into smaller bioactive peptides (the primary supplemental form of collagen) that are easily absorbed within the digestive tract before entering circulation (Iwai et al. 2005). Due to hydrolysis, collagen peptides do not possess the gelling properties of gelatine and are soluble in cold water. Currently, the sources of collagen peptides are bovine, porcine, marine and poultry hydrolysed collagen (León-López et al. 2019).

Research on collagen peptides and specific gelatine products (herein referred to as COL) has mainly focussed on the impact supplementation may have on bone and joint health, due to its role in regulating tendon and bone turnover (Viguet-Carrin et al. 2006; Ferreira et al. 2012). Even though findings are equivocal, there is compelling evidence that COL inhibits bone collagen breakdown and alleviates painful symptoms associated with degenerative joint conditions (García-Coronado et al. 2019). As a result, two review papers (Moskowitz 2000; Bello and Oesser 2006) concluded that COL could be used as a safe, therapeutic supplement in helping to manage symptoms associated with osteoarthritis and osteoporosis.

Due to the increased prevalence of long-term joint injuries and osteoarthritis from sport participation (Bennell et al. 2012), there is an emerging interest in therapeutic interventions to improve joint health. This includes research involving specific exercise protocols combined with COL to examine the impact on joint functionality during activity and recovery. It is likely that exercise would aid the benefits of COL due to the ‘mechano-transduction’ hypothesis, which proposes that mechanical loading of tendon tissue during exercise creates a signalling cascade in the tissue cells that increases production of matrix proteins and subsequent tendon hypertrophy (Svensson et al. 2016). In addition, collagen synthesis is likely to increase with of the co-ingestion of vitamin C, through its role in the hydroxylation of proline and lysine, both of which are essential in creating the collagen helix formation and intermolecular cross-linking (Paxton et al. 2010).

There has been an influx of research investigating the impact of COL and exercise; however, there is currently no consensus. Therefore, the purpose of this systematic review is to evaluate the effect of COL and exercise on joint function and athletic recovery, and gauge appropriate dosing strategies via collation and critique of the available literature. As such, the focus of the review is on the impact COL and exercise on parameters such as: joint function, muscle and joint injury recovery, body composition, muscle protein synthesis (MPS) and collagen synthesis.

## Methods

### Search strategy

Relevant published randomised controlled trials studying the effect of COL and exercise were identified in June 2020, using the following online databases: PubMed, Web of Science and CINAHL. The following keywords and terms were used: collagen ‘OR’ collagen supplementation ‘OR’ collagen peptide supplementation ‘OR’ gelatine/gelatin ‘AND’ exercise ‘OR’ athletes ‘OR’ recovery ‘OR’ strength performance ‘OR’ aerobic exercise ‘OR’ nutritional supplement. In addition, ‘clinical trial’ and ‘randomised controlled trial’ filters were applied to narrow our search. There was no restriction on the original language of the study and an automatic page translation to English was applied. All the retrieved studies were manually screened by the authors L.C. and M.K. for relevance. The studies were saved in a reference manager software (EndNote X9, Thomson Reuters©, New York, NY, USA). The study literature was reviewed using Preferred Reporting Items for Systematic Reviews and Meta-analyses (PRISMA) guidelines (Moher et al. 2009).

### Selection criteria

#### Inclusion criteria

Studies were included if they fulfilled the following criteria (i) had an exercise protocol or recorded physical activity; (ii) used different forms of COL, which may contain additional supplements or therapies; (iii) randomised, double-blinded, placebo-controlled or isonitrogenous supplement trials; (iv) participants over the age of 18 years; who were either athletic or recreationally active, had joint injuries, or had early signs of sarcopenia (class *I* or *II*).

#### Exclusion criteria

Studies were excluded if there was: (i) no exercise or a physical exertion test in the study protocol; (ii) participants had a degenerative joint disease, such as osteoarthritis; (iii) no blinding; (iv) no placebo control or similar isonitrogenous supplementation; (v) grey literature, such as seminar posters or only abstracts; (vi) in animals; (vii) participants below the age of 18 years.

### Data extraction

The data from the included articles were extracted by the lead author (M.K.) and was cross-checked for accuracy by a second author (L.C.). The following information was extracted from the final articles: author list, year of publication, study title, number of participants, participant characteristics, type of COL, dosage, supplementation length, exercise modality, exercise programme length, methods utilised, outcome measures, and main findings.

### Assessment of quality of methodologies of studies

The National Heart, Lung and Blood institute’s (NHLBI) quality assessment tool for controlled intervention studies was used to evaluate the methodological quality of studies included in this systematic review (https://www.nhlbi.nih.gov/health-topics/study-quality-assessment-tools). The studies were assessed using 14 questions cited in the NHLBI tool. For the methodological quality assessment, items were rated as 1 (meets criteria), 0 (does not meet criteria) or N/A (not applicable). The final score for the included article was calculated by the total sum of items scored, divided by the total number of items scored and expressed as a percentage. Two of the authors (M.K. and L.C.) independently assessed the quality of the studies, with any disagreements discussed until a consensus could be reached, before this a Cohen’s kappa score of 0.74 was reached indicating a substantial level of interrater reliability. The NHLBI tool does not have any set threshold for quality scores. Nonetheless, using general study quality assessment guidelines, studies were characterised as having either low (≤ 50%), good (51–75%) or excellent (> 75%) methodological quality (Field et al. 2020).

## Results

### Main search

**Fig. 1:**
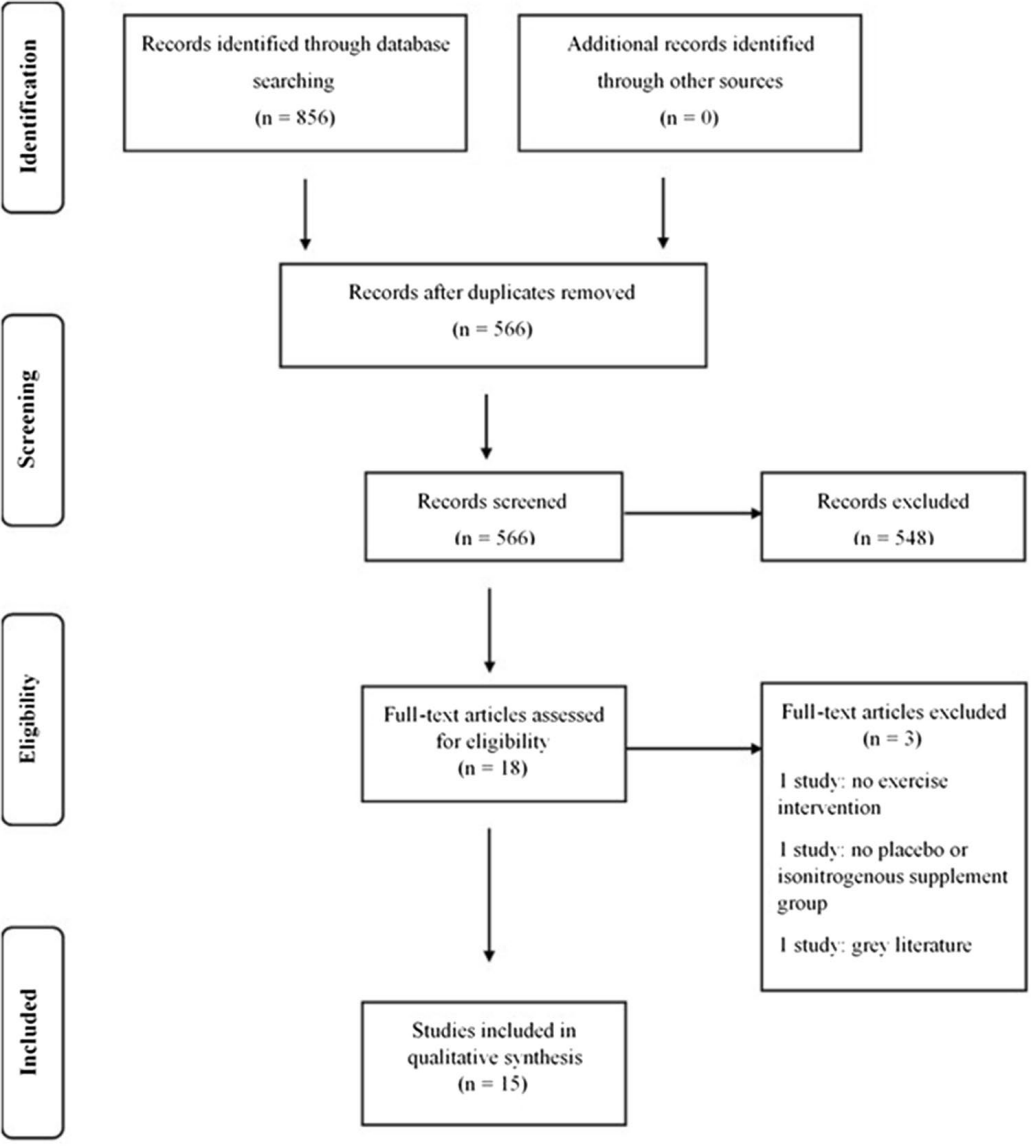
PRISMA flow diagram

Out of 856 studies identified, 15 studies were included in the final review (Fig. 1). Two hundred and ninety duplicate studies were removed and 566 study abstracts were screened using the EndNote X9 citation manager software; 18 studies then went through a full text check. Three studies were excluded as there was no placebo control (*n* = 1), only the abstract was available (*n* = 1) and did not have an exercise intervention (*n* = 1). All included studies were double-blinded and placebo-controlled or, as in two studies, provided an equivalent protein isonitrogenous control (Oikawa et al. 2020a, b). The main data and findings from these studies are summarised in Tables 1, 2, 3 and 4.

**Table 1:** Studies assessing effects of collagen supplementation on joint pain and recovery from joint injuries

Study	QACIS score	Participants	Type and dosage	Exercise modality	Outcome measures	Main findings
Clark et al. (2008)	78.57%	97 varsity team or club sport level athletes with activity-related joint pain (45 males, 52 females) (20 ± 1 years)	10 g/day collagen hydrolysate	Continued routine sporting activity for 24 weeks	VAS for joint discomfort Physician’s assessment of joint discomfort	↓ in joint pain with COL vs PLA ↓ joint pain at rest (*p* = 0.025), walking (*p* = 0.007), standing (*p* = 0.011), carrying objects (*p* = 0.014) and lifting (*p* = 0.018) ↓ in alternative therapies in COL vs PLA (12 vs 39 times, from baseline)
Lugo et al. (2013)	85.71%	55 participants with joint discomfort on doing physical activity (46 ± 2 years COL, 47 ± 2 years PLA)	40 mg/day of undenatured type II collagen derived from chicken sternum	Step-mill exertion test after 4 months	Knee flexion and extension to assess joint function	↑ in knee extension with COL vs PLA (*p* = 0.011) ↑ in length of pain-free strenuous exertion with COL from baseline (2.8 ± 0.5 min vs 1.4 ± 0.2 min; *p* = 0.019)
Zdzieblik et al. (2017)	92.86%	139 athletic participants (56 males, 83 females) (24 ± 0.3 years)	5 g/day of collagen peptides	Regular exercise, at least 3 h per week for 12 weeks	VAS for pain at rest and during activity Joint mobility through range of motion methods. Both evaluated by a physician	↓ in knee pain seen in both groups (*p* < 0.001), but reduction was more pronounced in COL group as compared to PLA (38.4 vs 27.9%, respectively) on VAS scale ↓ in alternative therapies in COL vs PLA (59 vs 40%, from baseline)
Dressler et al. 2018	71.43%	50 athletic participants (24 males, 26 females) (27 ± 9 years)	5 g/day of collagen peptides	3 home-based exercise sessions per week for 6 months (rope-skipping, squats and one-legged heel raises)	CAIT—a self-reported questionnaire FAAM-G questionnaire Ankle arthrometer to measure ankle stiffness	↑ in perceived ankle function in COL CAIT score ↑ by 5.28 ± 1.16 in COL (*p* < 0.001) No change in CAIT score was analysed in the PLA
Praet et al. (2019)	85.71%	20 participants with Achilles tendon symptoms (12 males, 8 females) (44 ± 8 years)	5 g/day of collagen peptides	Eccentric calf-strengthening programme performed daily for 6 months	VISA-A questionnaire Real-Time Harmonic Contrast Enhanced Ultrasound to measure tendon thickness	COL may ↑ the clinical benefits of a well-structured calf-strengthening and return-to-running program in Achilles tendinopathy patients ↑ VISA-A score in COL (12.6 points) vs PLA (5.3 pts), and ↑ (17.9 vs 5.9 pts) after crossing over
Average QACIS score	83%					

*VAS* Visual analogue scale, *COL* Collagen peptide supplementation, *PLA* Placebo, *CAIT* Cumberland Ankle Instability Tool, *FAAM-G* Foot and Ankle Ability Measure German version, *VISA-A* Victorian Institute of Sports Assessment–Achilles, ↑ increased, ↓ decreased

**Table 2:** Studies assessing the effects of collagen supplementation on body composition and muscle strength

Study	QACIS score	Participants	Type and dosage	Exercise modality	Outcome measures	Main findings
Zdzieblik et al. (2015)	92.86%	53 elderly sarcopenic men (72 ± 5 years)	15 g/day collagen peptides	12-week guided RT programme on fitness devices	DEXA scan One leg stabilisation test for SMC IQS	↑ in FFM, BM, SMC and IQS with COL and RT vs PLA (*p* < 0.05) FFM (+ 4.2 ± 2.3 kg COL vs +2.9 ± 1.8 kg PLA), IQS (+16.5 ± 13 Nm COL vs +7.3 ± 13 Nm PLA) ↓ in FM in COL (*p* < 0.05) FM (− 5.4 ± 3 kg COL vs – 3.5 ± 2 kg PLA)
Oertzen-Hagemann et al. (2019)	78.57%	25 recreationally active men (24 ± 3 years)	15 g/day of collagen peptides	3 times per week 12-week resistance training intervention with barbells	BIA Leg extension maximum involuntarily contraction Muscle biopsies for muscle proteome analysis	↑ in BM (*p* = 0.035), FFM (*p* = 0.014) and rowing (*p* = 0.025) in COL vs PLA ↑ in all parameters (BM, FM, FFM, squat, deadlift, bench-press, rowing and isometric strength) from baseline to the culmination (*p* ≤ 0.05), slightly more pronounced effects in COL 221 ↑ abundant proteins in COL vs 44 in PLA
Kirmse et al. (2019)	71.43%	57 young men (24 ± 3 years)	15 g/day of collagen peptides	3 times per week of resistance exercise training for 12 weeks	Strength testing via leg extension maximum voluntary contraction BIA for body composition testing Muscle biopsies for muscle fibre distribution and thickness analysis	↑ in FFM with COL (p < 0.05) ↔ in BFM with COL, but ↑ with PLA ((8.8 ± 3.2 vs 9.5 ± 3.0 kg, pre vs post) ↑ in isometric strength (*p* = 0.477), one-repetition maximum strength (*p* < 0.001) and type II muscle fibres (*p* < 0.001) in whole cohort from pre to post, but slightly more pronounced in COL
Jendricke et al. (2019)	85.71%	77 pre-menopausal women (38 ± 9 years COL, 42 ± 7 years PLA)	15 g/day of collagen peptides	3 times per week of resistance exercise training for 12 weeks	Body composition were determined by BIA Muscular strength by isometric strength testing	↑ in FFM with COL (*p* < 0.05) ↓ in BFM with COL (*p* < 0.05) ↑ in hand-grip (*p* < 0.05) and leg (p < 0.01) strength, ↔ in PLA
Average QACIS score	82%					

*RT* Resistance training, *DEXA* Dual-energy X-ray absorptiometry, *COL* Collagen peptide supplementation, *PLA* Placebo, *FFM* Fat free mass, *BM* Bone mass, *SMC* Sensory motor control, *IQS* Isokinetic quadricep strength, *FM* Fat mass, *BFM* Body fat mass, *BIA* Bioelectrical impedance analysis, ↑ increased, ↓ decreased, ↔ unchanged

**Table 3:** Studies assessing the effects of collagen supplementation on muscle soreness and recovery from exercise

Study	QACIS score	Participants	Type and dosage	Exercise modality	Outcome measures	Main findings
Lopez et al. (2015)	85.71%	8 recreationally active participants (6 males, 2 females) (33 ± 13 years COL, 26 ± 2 years PLA)	3 g/day of BioCell collagen supplement	UBC for 6 weeks after supplementation (day 43), redone after 3 days (day 46)	UBC test results to assess recovery of muscle strength, endurance and return of functional capacity Serum markers for muscle tissue damage PRS VAS for DOMS	↓ in the total number of repetitions performed on day 43 and 46, but PLA was 14% lesser than COL Decline in first set to subsequent seven sets in COL was 57.9% on day 43 and 57.8% on day 46 vs 72.2% on day 43 and 65% on day 46 in PLA PRS ↑ by 1.8 pts for COL and ↓ by 0.2 pts for PLA on day 46 (8.3 pts vs 7.3 pts on day 48, COL vs PLA; ES = 0.66) Plasma biomarkers CK and LDH ↓ with COL (↓ by 9.3 U/L in COL vs ↑ by 935.0 U/L in PLA)
Clifford et al. (2019)	71.43%	24 recreationally active males (24 ± 4 years COL, 25 ± 5 years PLA)	20 g/day of collagen peptides	150 drop jumps	Maximal isometric voluntary contractions CMJ Muscle soreness (200 mm VAS) Pressure pain threshold BAM+ Blood markers to gauge inflammation, bone turnover and muscle damage	↓ in muscle soreness with COL 48 h after exercise (90.42 ± 45.33 mm vs. PLA 125.67 ± 36.50 mm, ES = 2.64) ↑ in CMJ height recovery with COL vs PLA at 48 h (*p* = 0.05) COL had moderate benefits for the recovery of CMJ and muscle soreness but had no influence on inflammation and bone collagen synthesis (*p* > 0.05)
Average QACIS score	82%					

*UBC* Upper body resistance exercise stress challenge, *COL* Collagen peptide supplementation, *PLA* Placebo, *PRS* Perceived recovery scale, *VAS* Visual analogue scale, *DOMS* Delayed onset of muscle soreness, *ES* Effect size, *CK* Creatine kinase, *LDH* Lactate dehydrogenase, *CMJ* Countermovement jumps, *BAM+* Brief Assessment of Mood Adapted, ↑ increased, ↓ decreased

**Table 4:** Studies assessing the effects of collagen supplementation on collagen synthesis and muscle protein synthesis

Study	QACIS score	Participants	Type and dosage	Exercise modality	Outcome measures	Main findings
Shaw et al. (2017)	91.67%	8 recreationally active men (27 ± 6 years)	5 or 15 g/day of vitamin C enriched gelatine	6 min of rope-skipping 3 times a day for 3 days	Engineered ligaments were used to analyse the functional effect of COL	↑ in collagen synthesis with 15 g COL (153% from baseline, *p* < 0.05) vs 5 g/day gelatine group (59.2%) and PLA (53.9%)
Lis and Baar (2019)	71.43%	10 recreationally active men (23 ± 5 years)	15 g/day collagen hydrolysate or gelatine or gummy containing both	6 min jump rope	Blood samples to assess collagen synthesis	COL may improve collagen synthesis when taken 1 h prior to exercise. But large variability in results led no statistically significant treatment
Oikawa et al. (2020a)	85.71%	11 recreationally active participants (5 males, 6 females) (24 ± 4 years)	60 g/day of collagen peptides (20 g post-exercise and 40 g pre-sleep)	4 × 4 min cycling at 70% of peak power output for 3 days	Deuterated water, muscle biopsy and blood samples to assess MPS TQRS and KSS to assess sleep quality	↑ in MPS with LA vs COL (*p* < 0.01) ↔ in sleep quality with LA or COL
Oikawa et al. (2020b)	92.86%	22 healthy older women (69 ± 3 years)	30 g/day of collagen peptides	Resistance exercise performed twice during the 9-day study period	Saliva swab and muscle biopsy to gauge acute and long-term MPS	↑ in MPS with WP at rest and with exercise (*p* < 0.01, acutely and *p* < 0.0001, long term) ↑ MPS only with exercise in COL (*p* < 0.01) and no long-term effects
Average QACIS score	85%					

*COL* Collagen peptide supplementation, Placebo, *MPS* Muscle protein synthesis, *LA* Lactalbumin, *TQRS* Total Quality Recovery Scale, *KSS* Karolinska Sleepiness scale, *WP* Whey protein, ↑ increased ↔ decreased

### Included studies

Fifteen studies were included, of these 8 used collagen peptides or collagen hydrolysate in doses of 5–15 g/day (Clark et al. 2008; Zdzieblik et al. 2015; Zdzieblik et al. 2017; Dressler et al. 2018; Praet et al. 2019; Oertzen-Hagemann et al. 2019; Kirmse et al. 2019; Jendricke et al. 2019), 1 used 20 g/day of collagen peptides (Clifford et al. 2019), 1 used 30 g/day of collagen hydrolysate (Oikawa et al. 2020b), 1 used 60 g/day of collagen hydrolysate (Oikawa et al. 2020a), 2 used gelatine in doses of 5 g/day and 15 g/day (Shaw et al. 2017; Lis and Baar 2019) and 2 used original supplements containing 40 mg/day and 3 g/day of collagen peptide (Lugo et al. 2013; Lopez et al. 2015) (see Tables 1, 2, 3 and 4 for more detail). The supplements were provided in either a capsule or powdered form, consumed with water.

All the trials were conducted between 2005 and 2019. There were a total of 656 participants, with 325 males and 276 females; one study did not report the number of male and female participants (Lugo et al. 2013). Twelve studies had recreationally active participants (average age: 30 ± 10 years) who experienced joint-related discomfort (Clark et al. 2008; Zdzieblik et al. 2017; Dressler et al. 2018; Praet et al. 2019; Oertzen-Hagemann et al. 2019; Kirmse et al. 2019; Clifford et al. 2019; Oikawa et al. 2020a; Shaw et al. 2017; Lis and Baar 2019; Lugo et al. 2013; Lopez et al. 2015), 2 studies were in an elderly population; 1 in men experiencing onset of sarcopenia (age 72 ± 5 years) (Zdzieblik et al. 2015) and the other in healthy, older women (age 69 ± 3 years) (Oikawa et al. 2020b), and 1 study was in untrained pre-menopausal women (age 40 ± 8 years) (Jendricke et al. 2019). Twelve studies reported funding sources, but none of the studies reported conflicts of interest.

### Study outcomes

Study outcomes were categorised into different topics due to the range of effects COL and exercise may have. Studies were categorised into four topics: (i) joint pain and recovery from joint injuries, (ii) body composition, (iii) muscle soreness and recovery from exercise, and (iv) MPS and collagen synthesis.

#### Studies assessing effects of collagen supplementation on joint pain and recovery from joint injuries

Five studies (Table 1; average quality score (QS): 83%) observed positive effects of COL in reducing joint discomfort and knee pain, improving ankle and knee functionality, and accelerating recovery from Achilles tendinopathy. The studies used 40 mg, 5 g and 10 g per day doses of COL. One study was conducted over 3 months (Zdzieblik et al. 2017), one over 4 months (Lugo et al. 2013), and three studies over 6 months (Clark et al. 2008; Dressler et al. 2018; Praet et al. 2019).

Clark and colleagues (2008) noted an effect size (ES) of 0.36 for ‘joint pain when walking’, but in a knee arthralgia sub-group, the ES was slightly higher (0.45), indicative of a small beneficial effect of COL (< 0.2 = weak effect; > 0.8 = strong effect). In addition, Zdzieblik and colleagues observed a moderate effect of COL (Cohen’s *d* = 0.30) in the ‘pain during activity’ parameter. An improvement in knee extension range (*p* = 0.011) and increase in pain-free exercise duration (*p* = 0.019) was also observed with undenatured type *II* collagen (UC-*II*; Lugo et al. 2013). Dressler et al.’s (2018) observed an improvement in subjective ankle stability in the Cumberland Ankle Instability Tool (CAIT; *p* < 0.001) during the 3-month follow-up. Similarly, in Achilles tendinopathy patients, an increase in Victorian Institute of Sports Assessment–Achilles questionnaire scores were noted in the COL groups (12.6 and 17.7 points), whereas the PLA groups only had slight change (5.3 and 5.9 points; Praet et al. 2019). However, microvascularity decreased equivalently in both COL and PLA groups.

#### Studies assessing the effects of collagen supplementation on body composition

Four studies (Table 2) evaluated the effects of collagen supplementation on body composition and/or muscle strength, using collagen or a placebo (PLA) coupled with a resistance training exercise programme (QS = 82%). One study was conducted in elderly sarcopenic men (15 g/day COL for 3 months; Zdzieblik et al. 2015), two studies were in recreationally active men (15 g/day COL for 3 months; Oertzen-Hagemann et al. 2019; Kirmse et al. 2019) and one study was in untrained pre-menopausal women (15 g/day COL for 3 months; Jendricke et al. 2019).

Jendricke and colleagues (2019) found a greater increase in fat free mass (FFM) (*d* = 0.55), decrease in body fat percentage (BF) (*d* = 0.54), and an increase in hand-grip strength (*d* = 0.63), suggesting a medium effect size with COL. Comparably, Kirmse and colleagues (2019) noticed a significant increase in FFM with COL (*p* < 0.05), and there was a significant increase in body fat mass with PLA (*p* < 0.05). Another study in recreationally active men found a significant decrease in body mass (*p* = 0.035) and increase in FFM (*p* = 0.014) with COL, whereas no change was observed in fat mass (FM) for either COL or PLA (Oertzen-Hagemann et al. 2019). While elderly sarcopenic men showed an overall improvement with resistance training, more pronounced changes were seen with COL (*p* < 0.05) (Zdzieblik et al. 2015). However, though there were moderate changes with COL in recreationally active men and untrained pre-menopausal women, they were not as prominent as the previous study (Zdzieblik et al. 2015).

#### Studies assessing the effects of collagen supplementation on muscle soreness and recovery from exercise

Two studies (Table 3) assessed the effect collagen supplementation had on subsequent exercise performance and recovery from muscle soreness (QS = 82%; Lopez et al. 2015; Clifford et al. 2019). The studies were conducted in recreationally active men. One study used 3 g/day COL for 6 weeks (Lopez et al. 2015) and the other used 20 g/day COL for 7 days (Clifford et al. 2019), prior to their respective muscle damaging exercise protocols.

A moderate effect size on perceived recovery (ES = 0.66) and discomfort (ES = 0.64), and a strong effect for ‘pain with movement’ (ES = 1.12) was observed following COL (Lopez et al. 2015). Similarly, a large effect size (ES = 2.0–4.0) on muscle soreness with COL was detected 24 h (ES = 2.40) and 48 h (ES = 2.64) post-exercise by Clifford and colleagues (2018).

#### Studies assessing the effects of collagen supplementation on muscle protein synthesis and collagen synthesis

Two studies (Shaw et al. 2017; Lis and Baar 2019) assessed the effects of COL on collagen synthesis with different forms or doses of collagen and two studies (Oikawa et al. 2020a and b) investigated the effects of collagen compared to a different form of protein on MPS (QS = 85%). Shaw and colleagues (2017) observed a significant increase in collagen synthesis markers (N-terminal peptide of procollagen—PINP) following ingestion of 15 g COL enriched with vitamin C (0 ng/ml vs 30 ng/ml, PINP levels, baseline vs 4 h), consumed 60 min prior to exercise. Two studies observed a higher increase in muscle protein synthesis with whey protein and lactalbumin vs COL (Oikawa et al. 2020a, b). The results from these studies are detailed in Table 4.

## Discussion

The purpose of this systematic review was to examine the effects of COL on exercise performance, recovery, and rehabilitation in the elderly, and elite and recreational athletes. The most prominent effects of COL were observed on joint function and recovery from joint injuries.

### Effects of collagen supplementation on joint function and recovery from joint injuries

All five studies reported beneficial effects of COL in reducing joint pain, improving joint function, increasing the length of pain-free strenuous exertion, and reducing the need for alternative therapies, especially when combined with an exercise rehabilitation programme (Table 1). Both Clark and colleagues (2008) (10 g/day COL) and Zdzieblik et al. (2017) (5 g/day COL) observed that COL led to a decrease in activity-related joint discomfort and use of alternative therapies to manage pain (PLA alternative therapy use 3.25-fold higher than COL in the former study and COL resulted in a 59% decrease of therapies in the latter). Interestingly, even though Zdzieblik et al. (2017) supplemented for 12 weeks, it was still effective in alleviating joint symptoms. This is in contrast to Clark and colleagues (2008), who did not see a statistically significant improvement until the final visit at 24 weeks, implying that it may take ≥ 3 months to realise the benefits of COL. These results also suggest that 5 g/day COL may be as effective as 10 g/day COL in alleviating pain during activity for athletes, in the absence of a degenerative joint disease. A possible explanation for the reduction in joint pain may be that COL increases type *I*,* II*,* IV* collagen, proteoglycan, and elastin synthesis in the articular cartilage, possibly reducing tissue damage and decreasing pain (Oesser and Seifert 2003; Schunck and Oesser 2013). Collagen peptide supplementation may also aid formation of ECM molecules leading to increased firmness of the connective tissue, and downregulation of matrix metalloproteinases that degrade ECM collagen proteins (Schunck and Oesser 2013). Moreover, collagen peptides may possess anti-inflammatory properties, as glycine can inhibit pro-inflammatory cytokine release (e.g. interleukin-6; Hartog et al. 2013). However, more human studies are required to better understand the regulatory mechanisms of COL.

Young athletes had improved ankle function with COL, citing a lower feeling of the ankle ‘giving away’ and a decrease in reoccurrence of ankle injuries after suffering from chronic ankle instability (possibly having clinical applicability; Dressler et al. 2018). Nevertheless, the baseline CAIT scores were lower for COL than PLA, which may have led to difference in ankle function improvements in the target group. Similarly, Praet and colleagues (2019) coupled COL with an eccentric bi-daily calf strengthening and a return-to-running exercise protocol in athletes suffering from Achilles tendinopathy. The participants were able to return to running after the treatment but did not reach pre-injury levels within the duration of the study. The COL used contained 22% glycine, which is known to enhance collagen matrix organisation strength, reduce inflammation and influence tenocyte metabolism in tendons (Vieira et al. 2018). The eccentric training protocol used may have also improved the tendon structure and reduced neovascularization (associated with tendinosis; Ohberg and Alfredson 2004). Indeed, eccentric training can elicit a transformation in the ECM composition of skeletal muscles through the remodelling of endomysial type *IV* collagen (Mackey et al. 2004).

In a study by Lugo et al. (2013), UC-*II* derived from chicken sternum increased pain-free exercise duration (2.8 min vs 1.4 min, COL vs baseline) and improved knee extension range of motion (81.0 ± 1.3° vs 73.2 ± 1.9°, COL vs baseline). However, no significant changes were observed in either exercise duration or knee extension range with PLA. The beneficial effects of COL in this study could be due to the activated T regulatory cells specific to UC-*II*. Type *II* collagen releases anti-inflammatory cytokines (interleukin-10 and transforming growth factor-β) that have the potential to counter the pro-inflammatory cascade associated with strenuous physical exertion, creating a shift towards ECM replenishment by the chondrocytes (Lugo et al. 2013). Therefore, supplementing even 40 mg/day of UC-*II* may have the potential to improve joint functionality and range of motion. Overall, COL, coupled with a rehabilitative exercise protocol, may accelerate recovery from joint injuries and improve joint function, possibly via its anti-inflammatory properties, or effects on ECM regeneration, and collagen synthesis in cartilage and tendons.

### Effects of collagen supplementation on body composition

Out of the four studies assessing changes in body composition and muscle strength, Zdzieblik et al. (2015) found COL complemented with a guided resistance training programme induced significant changes in elderly sarcopenic men (class *I* or *II*). There was an increase of over 5 kg of FFM and a decrease of 6 kg in FM with COL. Whereas in PLA, FFM increased by 3 kg and FM decreased by 4 kg, likely due to the resistance training programme (Zdzieblik et al. 2015). Though changes were seen in both groups, the effects were more noticeable in the COL group (*r* 0.72; *p* < 0.001 vs *r* 0.55; *p* < 0.003, correlation coefficient COL vs PLA). In addition, the outcomes were not as pronounced in pre-menopausal women, with only a 1.8% increase in FFM (Jendricke et al. 2019) or in recreationally active young with two studies noting a 2 kg increase in FFM (Oertzen-Hagemann et al. 2019; Kirmse et al. 2019). Kirmse et al. (2019) and Jendricke et al. (2019) both observed a significant increase in FFM with COL, with the latter observing a decrease in FM in pre-menopausal women (− 1.5 ± 1.7 kg COL vs + 0.7 ± 1.6 kg in PLA). The changes in FFM are possibly attributed to an increase in surrounding connective tissue, as there was no difference in fibre cross-sectional area (fCSA) hypertrophy in either group (Kirmse et al. 2019). Previous studies have also observed an increase in ECM synthesis in connective tissue with COL supplementation (Schunck and Oesser 2013). For changes in FM, COL has shown to reduce body weight gain and adipocyte enlargement (Chiang et al. 2016). However, the BF losses reported in elderly sarcopenic men was likely to be higher, as participants had ~ 30% BF (Zdzieblik et al. 2015), whereas participants in Kirmse et al. (2019) had ~ 11% BF at baseline.

Using skeletal muscle proteomics, Oertzen-Hagemann et al. (2019) observed that COL induced a higher increase in proteins (such as myosin proteins, actin-binding proteins and tropomyosins) related to resistance training adaptations, likely due to the high hydroxyproline-peptide concentration in collagen peptide supplements as observed previously (Kitakaze et al. 2016). Furthermore, a higher increase in myotilin, a muscle Z-disk protein, which is an important marker for myofibril remodelling post-exercise, was observed in the COL group. The higher upregulation of proteins with resistance training and COL indicates a deeper effect on skeletal muscle proteomes as compared to resistance training solely. Adaptations in the ECM seemed to have occurred largely due to the resistance training programme and may have been independent of COL as similar proteins affecting the ECM were upregulated in PLA (Kjaer et al. 2006).

Collagen supplementation combined with resistance training elicited moderate improvements in body composition. The increase in FFM is purportedly due to COL’s effect on the surrounding connective tissue, and not myofibrillar protein, as there were no changes observed in fCSA hypertrophy.

### Effects of collagen supplementation on muscle soreness and recovery from exercise

To date, two studies have investigated the impact of COL on muscle soreness and recovery from strenuous exercise (Lopez et al. 2015; Clifford et al. 2019). Collagen peptides derived from chicken sternal cartilage seemed to attenuate decrements in bench-press performance, improve recovery (8.3 vs 7.3 points, COL vs PLA on a perceived recovery scale), and reduce symptoms of delayed onset of muscle soreness (58 vs 72%, decrease in performance COL vs PLA; Lopez et al. 2015). Plasma biomarkers for muscle damage and inflammation were also lower in the COL group. A higher tolerance for repeated high-intensity resistance exercise protocol in the intervention group was observed, demonstrating that COL may accelerate the protective adaptation of the ‘repeated bout effect’, allowing for enhanced musculoskeletal recovery by possible ECM remodelling (Lopez et al. 2015). In contrast, Clifford et al. (2019) found no influence of COL on markers of inflammation and bone collagen synthesis, although COL was found to reduce muscle soreness by around 4.1–5.4 mm post-exercise on a visual analogue scale. As the study assessed inflammation and bone collagen turnover through blood samples, the mechanisms behind the positive changes observed could not be thoroughly explained. A larger sample size using muscle biopsies and additional biomarkers for connective tissue turnover is required in future studies to get a greater understanding of how COL might influence recovery.

Previously, COL has reduced joint and muscle pain in recreational athletes (Clark et al. 2008; Zdzieblik et al. 2017) and aided recovery in active individuals (Lopez et al. 2015). Recently, a study found that whey protein can stimulate collagen synthesis in muscle after resistance training (Holm et al. 2017). Though collagen has a different amino acid profile from whey protein, it will be interesting to gain an understanding into the adaptations occurring with COL.

### Effects of collagen supplementation on collagen synthesis and muscle protein synthesis

Four studies assessed the effects of COL on collagen synthesis and muscle protein synthesis. Shaw et al. (2017) found that collagen synthesis increased and remained elevated for 72 h, with 15 g/day COL enriched with vitamin C as compared to a 5 g/day dose and PLA. The amino acid content of blood peaked 1 h after 15 g COL consumption (increase of 376 mmol/L in glycine, and 162 mmol/L of proline vs baseline) and 30 min after 5 g COL, providing important information regarding exercise timing in accordance with the collagen dose. The 15 g/day COL augmented collagen synthesis in the recovery period after exercise as seen by the increase in bone collagen synthesis markers (PINP) (153% increase in PINP with 15 g COL, vs 59.2% increase with 5 g COL and 53.9% increase with PLA). This indicates that the improved collagen synthesis with 15 g/day COL coupled with an intermittent exercise protocol, consumed 60 min prior to exercise, may improve tissue repair and help prevent injuries.

In addition, the presence of vitamin C promotes hydroxyproline formation (Pinnell et al. 1987) and cultivates collagen cross-linking (Levene et al. 1972), making it essential for collagen synthesis.

Interestingly, only Shaw et al. (2017) found that bone collagen synthesis markers (PINP) increased significantly with COL (15 g dose only) whereas, Clifford et al. (2019) and Lis and Baar (2019) did not observe any changes in PINP following 20 g/day COL and varied doses (15 g gelatine enriched with vitamin C, 15 g hydrolysed collagen and a 15 g gummy containing 7.5 g gelatine and 7.5 g hydrolysed collagen), respectively. Lis and Baar (2019) suggested that they did not see significant changes due to high variability of the PINP testing kit used. The addition of vitamin C may interfere with the Enzyme-Linked Immunosorbent Assay (ELISA) kit used in this study, which is in contrast to Shaw and colleagues results (2017). This could be due to the difference in collagen supplements (gelatine and hydrolysed collagen) and the blood clotting time (20 min) used by Lis and Baar (2019), whereas Shaw et al. (2017) used only gelatine and a 2-h clotting time. Heat and a longer waiting period may degrade vitamin C, reducing the variability of the ELISA reaction. Hence, a more reliable outcome measure (possibly mass spectrometry), and setting testing protocols for vitamin C inclusion, are needed to conclude which form of COL may be most effective.

Two studies compared the effect that COL has on MPS (i) 30 g/day whey protein vs 30 g/day COL (Oikawa et al. 2020a) and (ii) 60 g/day lactalbumin vs 60 g/day COL (Oikawa et al. 2020b), finding a greater increase in MPS with whey protein (0.173 ± 0.104% whey protein vs 0.020 ± 0.034% COL, after exercise) and with lactalbumin (13 ± 5% higher than COL). Though the supplements were isonitrogenous, the plasma leucine concentration was 87% higher with lactalbumin and 5.5 times higher in whey protein than COL, which is the likely reason for the increased MPS (Devries et al. 2018). Indeed, lactalbumin and whey protein may be considered higher quality proteins due to AA profile (high amounts of leucine and tryptophan), digestibility and bioavailability, earning each a Protein Digestibility-Corrected Amino Acid Score of 1. Whereas, COL has a PDCAAS score of 0, as it lacks the essential AA tryptophan (Phillips 2017). Protein quality may impact skeletal muscle adaptations and recovery, hence lactalbumin might be better suited for supplementation following exercise if the main priority is hypertrophy. Interestingly, whey protein induced a higher MPS response at rest and with exercise acutely and up to 4 h later, whereas COL only elevated MPS levels acutely with exercise (Oikawa et al. 2020a). Intriguingly, Oikawa et al. (2020a) found no differences for muscle collagen protein synthesis following participants consuming either COL or whey protein, with a significant increase found as a result of exercise. Potentially indicating that total protein and amino acid profile may be more influential than type of protein on MPS and muscle collagen protein synthesis. However, further studies like these are necessary to assess different protein sources, doses, and exercise forms in a variety of populations to test their efficacy.

## Practical implications

Based on the studies collated in this review, collagen has the potential to reduce joint pain and improve joint functionality, especially when complemented with a rehabilitative exercise protocol. Collagen supplementation also increased pain-free time to exertion and collagen synthesis; hence, 5 g–15 g/day doses of COL when taken at least 1 h prior to exercise, for over 3 months, may aid in reducing functional joint pain and improving muscle recovery. As the beneficial effects of COL appear to take effect after three months or longer, athlete and participant compliance with the supplementation period is crucial. Prolonged use of collagen is deemed to be safe, with none of the studies within this review reporting any adverse effects of COL, even at higher doses (60 g/day) or different supplement forms. However, other higher quality protein sources, such as whey protein may be more beneficial for MPS, and therefore, muscle hypertrophy.

Traditionally, collagen is derived from animal bone and cartilage, but now vegan and vegetarian forms of COL are becoming more available (synthesised from genetically modified yeast and bacteria), making it accessible to a larger population. However, none of the collagen supplements reviewed in this paper was from vegetarian/vegan sources, and their efficacy cannot be assured until further research is conducted.

## Future research directions

As females are more prone to connective tissue injuries than males, it is critical to have more studies assessing the effects of COL on females. The increased risk of injuries in females is due to an attenuated tendon hypertrophy response, lower tendon collagen synthesis rate immediately after exercise and increased oestrogen levels which may reduce the mechanical strength and stiffness of tendons and ligaments (Magnusson et al. 2007).

Prevalence of bone-tendon injuries such as “jumper’s knee” is relatively high in the athletic population and can negatively impact an athlete’s career (Lian et al. 2005). Determining COL’s impact on recovery from such injuries may provide valuable information for improving recovery time.

Overall, there was a mixed consensus on COL mechanisms, and more controlled studies with precise outcome measures such as biochemical analysis, engineered human ligaments and muscle biopsies are required. Computed tomography, magnetic resonance imagery and ultrasonography should be included to directly measure changes in joint cartilage and tendon dimensions (Hayes et al. 2019).

## Limitations

The studies included in the review have some methodological concerns, such as differences in baseline characteristics that may alter the results, and a lack of consistency in outcome measures. Certain criteria, such as the reporting of outcome measure concealment from participants and researchers, and the method of randomisation, was often not reported. In addition, as unpublished studies (e.g. conference abstracts, presentations) were not included, and most studies were funded commercially (*n* = 13), the possibility of publication bias arises. Nonetheless, 11/15 studies included were regarded as having an excellent methodological quality score.

## Conclusions

- Strong evidence of 5–15 g/day dose of COL in improving joint pain and functionality. However, further research is required to understand the exact adaptive mechanisms.
- Changes in body composition and strength with 15 g/day COL and resistance training were not as prominent in young recreationally active participants as they were in elderly sarcopenic men.
- Exercise and vitamin C seemed to aid collagen synthesis. 15 g/day COL was more effective than 5 g/day COL in elevating collagen synthesis, hence 15 g/day may be a more effective dose. COL should be consumed prior (~ 60 min) to exercise to maximise collagen synthesis.
- Muscle recovery had a modest but significant improvement with COL.

## Data Availability

Not applicable.
